# Birth size, school performance and family social position: a study of 650,000 children

**DOI:** 10.1038/s41390-023-02757-1

**Published:** 2023-07-29

**Authors:** Karri Silventoinen, Juha Luukkonen, Mikko Myrskylä, Pekka Martikainen

**Affiliations:** 1 Population Research Unit, Faculty of Social Sciences, University of Helsinki, Helsinki, Finland; 2 Research Institute of Human Development, Kyoto International Social Welfare Exchange Centre, Kyoto, Japan; 3 Max Planck Institute for Demographic Research, Rostock, Germany; 4 Max Planck—University of Helsinki Center for Social Inequalities in Population Health, Rostock, Germany; 5 Max Planck—University of Helsinki Center for Social Inequalities in Population Health, Helsinki, Finland

## Abstract

**Background:**

Low birth weight (BW) is associated with lower cognitive functioning, but less is known of these associations across the full range of the BW distribution and its components. We analyzed how BW, birth length (BL) and birth ponderal index (BPI, kg/m^3^) are associated with school performance and how childhood family social position modifies these associations.

**Methods:**

Medical birth records of all Finnish children born in 1987–1997 were linked to school performance records at 16 years of age (*N* = 642,425). We used population averaged and within-siblings fixed-effects linear regression models.

**Results:**

BL showed a linear and BW a curvilinear association with school performance whereas for BPI the association was weak. The strongest association was found for BL explaining 0.08% of the variation in school performance in boys and 0.14% in girls. Demographic, gestational and social factors partly explained these associations. Similar but weaker associations were found within sibships. The association of BL with school performance was stronger at lower levels of family social position.

**Conclusion:**

BL shows a linear association with school performance and can explain more school performance variation than BW. At the population level, BL can offer useful information on intrauterine environmental factors relevant for cognitive performance.

**Impact:**

- Birth length is linearly associated with school performance in late adolescence and explains a larger proportion of school performance variation than birth weight.
- The association between birth length and school performance is stronger in families with lower socio-economic position.
- At the population level, birth length can offer information on the intrauterine environment relevant for later cognitive performance.

## Introduction

Childhood family background strongly influences the formation of adult social position,^1^ and education plays the most important mediating role when parental social resources are transmitted to the next generation.^2^ However, the effect of the childhood environment on social position can already have prenatal origins. Birth weight (BW), the most common indicator of the prenatal environment, is positively associated with educational performance in childhood^3^ and earnings in adulthood.^4^ Cognitive ability is most likely an important contributor to these associations since low BW is associated with lower IQ from childhood to adulthood^5^ and even with a smaller brain volume when compared to average BW children.^6^ However, the neurocognitive consequences of BW are not limited only to low BW since the association between BW and IQ both in childhood^7^ and adulthood^8^ can also be found across the total variation of BW. This suggests that BW can provide information on the prenatal environment relevant for further cognitive development across the full range of BW values. This interpretation is supported by studies showing that genetic variants of BW are not associated with IQ^9^ or academic performance^10^ in Mendelian randomization studies, thus suggesting that these associations are not causal but rather reflect the role of the prenatal environment.

When evaluating the role of BW as an indicator of the prenatal environment, it needs to be recognized that BW can reflect prenatal risk factors differently according to the social characteristics of the family. The variation of birth size is affected by both genetic and environmental factors.^11^ It is thus possible that BW more robustly reflects the intrauterine environment in families with low socio-economic position (SEP), whereas in families with higher SEP, it may be more affected by genetic factors not associated with cognitive development of offspring. It is also noteworthy that BW is a combination of birth length (BL) and body fatness, typically measured in neonates using the ponderal index, which can differently reflect the intrauterine environment. BL is not as widely used as BW in large-scale studies, but there is still convincing evidence that BL is positively associated with neurocognitive outcomes.^12^ Studies analyzing birth ponderal index (BPI) and later cognitive performance are rare. However, there is evidence that high maternal pre-pregnancy body mass index (BMI) is associated with increased BW and body fatness^13^ as well as delayed neurobehavioral development of offspring.^14^ BPI is also positively associated with adult BMI,^15^ which further shows a negative association with IQ in early adulthood.^12^ Thus, we can speculate that BL and BPI are associated with later cognitive performance in opposite directions, which can explain the leveling off or even decrease of the association between BW and cognitive performance at the highest level of BW.^16^ All these associations may also differ according to family SEP.

Since studies analyzing how different indicators of birth size are associated with school performance according to family SEP are rare, we studied this issue using a large population-based longitudinal cohort of children. We used three indicators of body size at birth (BW, BL and BPI) and three family SEP indicators (maternal education, paternal education and household income). We present the following study hypotheses: (i) BL shows a positive and BPI a negative association with school performance, contributing to an expected curvilinear association between BW and school performance; (ii) adjusting the results for other risk factors measured directly or indirectly by comparing full siblings and twins diminishes these associations; and (iii) these associations are stronger in low SEP families as compared to high SEP families, indicating a stronger influence of the prenatal environment.

## Data and methods

Supplementary fig. 1 presents the flow diagram of our study cohort. Our baseline data included all children born in Finland during the years 1987–1997 (*N* = 692,058). BW (kg) and BL (cm), as well as Apgar score at 1 min after the delivery, were measured in the hospital and made available for us through the National Medical Birth Register covering all live births in Finland. Based on this register, we also obtained information on gestational age, previous pregnancies and maternally reported smoking during the pregnancy (did not smoke, quit smoking during the first trimester, smoked and missing). We excluded children having missing information on BW or BL (*N* = 7017) or other birth-related measures (*N* = 2649) from the analyses. We used BPI calculated by dividing BW in kilograms (kg) by the cube of body length in meters (m^3^) as a measure of relative weight. Information on maternal and paternal education (basic, secondary and tertiary), the mother’s birth year and child’s birth year was obtained from the National Population Register. Household income after tax based on information on different income sources of all household members was obtained from the tax officials and classified in yearly quintiles. Children without information on family SEP were excluded (*N* = 3186).

In Finland, all children start the compulsory 9-year primary school at the age of 7, and thus are at the age of 16 at the end of primary school. The school grade in the final class of primary school was available from the Admission Register for Higher Education for all children who applied to secondary education. We used the mean of grades of all school subjects, which can range from 4 to 10. Children who did not start secondary education, had not finished primary school or died before their 16th birthday were excluded from the data (*N* = 32,781). All registers were linked using unique personal identification numbers by Statistics Finland and then delivered to the research group after removing personal identification codes. Our final study cohort included 646,425 children (49% girls; 93% of the original cohort). However, we had outliers for BL (<40 cm or >60 cm; *N* = 2098) and BPI (<20 kg/m^3^ or >40 kg/m^3^; *N* = 1049) which were removed from these analyses.

First, we analyzed the associations between birth size and school performance at the individual level using population-averaged regression models. In these analyses, we removed 16,161 twins or higher order multiples since they had, on average, lower BW, BL and BPI, but better school performance than singletons (Supplementary Table 1). We started these analyses by testing the linearity of these associations by fitting a squared term for each birth size indicator into a model that already included a linear term. For BW, the squared term was statistically significant for boys and girls (*p* < 0.00001). This was because the association leveled off at the higher end of BW distribution (Supplementary Table 2). For BL, the squared term was not statistically significant in boys (*p* = 0.6936) or girls (*p* = 0.6265), whereas for BPI, the squared term was statistically significant for girls (*p* = 0.0211) but not for boys (*p* = 0.1625). However, since even for girls the association did not strongly diverge from a linear association (Supplementary Table 2 and visual inspection) and the squared term was statistically significant mainly because of our very large sample size, we used only linear terms for BL and BPI for both sexes in the further analyses. In our statistical models, we standardized the indicators of birth size (mean of 0 and standard deviation of 1) and added the number 10 to guarantee that all values were positive, allowing us to correctly estimate the quadratic effects. Because of the strong correlations between BL, BW and BPI, we estimated all models separately for these indicators to avoid multicollinearity.

We started the individual-level analyses by estimating the associations between the three birth size indicators and school performance and studying how these associations were attenuated by other birth- and family-related factors. We adjusted the models firstly for demographic factors (birth year, maternal age at the time of delivery and parity), secondly for gestational-related risk factors (maternal smoking during pregnancy, gestational age, square of gestational age and Apgar score), and finally for the family SEP (maternal and paternal education and household income). We then continued these analyses by studying whether the associations between the birth size indicators and school performance differed by family SEP by stratifying the models by each indicator of family SEP. In these interaction analyses, we used the original classification for maternal and paternal education, but for family income we pooled quintiles 2–4 as the middle category.

After the individual-level analyses, we conducted analyses within full same-sex sibships and co-twins. Previous research has demonstrated that both family environment and genetic factors have an influence on education.^17^ Since full-siblings share their childhood family and 50% of genetic variation, using within-family methods we can control for the influence of early family environment and also genetic influences, partially. We assumed that if the family environment or genetic factors affect the association between birth size and school performance, the associations should be reduced or disappear in these within-family analyses. Co-twins optimally share their family postnatal environment, and further monozygotic twins are genetically identical. There can also be considerable variation in the prenatal environment between twin siblings in terms of sharing or not sharing the placenta or amniotic sac.^18^ However, because only register-based information was available, we could not distinguish between monozygotic and same-sex dizygotic twins. Together, we had 55,063 families with at least two brothers and 51,915 families with at least two sisters. For the twin-pair analyses, we had 2352 male and 2271 female complete same-sex pairs. These within- family analyses were conducted using fixed-effects linear regression models. In practice, this model creates a dummy variable for each family, thus removing all between-family variation from the associations between birth size and school performance.^19^ We adjusted these models first for demographic factors and then gestational-related risk factors to test whether within-family variation in them can modify the associations found. All analyses were conducted by the Stata/MP 17.0 for Windows statistical software (StataCorp, College Station, TX). Since the nonlinear associations are difficult to interpret only based on parameter estimates (the linear term and the squared term), we calculated marginal effects based on estimated parameters for the most important BW results and presented them as figures.

The study has been approved by Statistics Finland Board of Statistical Ethics (TK-53–1490–18) and the Social and Health Data Authority Findata (THL/2180/14.02.00/2020), which deemed exempt from informed consent when awarding the permission to use the data. The register data were originally collected for administrative and statistical purposes. The legal basis for processing this kind of personal information is scientific research as stated in the Finnish Personal Data Act (523/1999) and the EU General Data Protection Regulation. Use of the data was regulated and permitted by the Act on Secondary Use of Social and Healthcare Data (552/2019) and the Finnish Statistics Act (280/2004).

## Results

Table 1 presents the descriptive statistics of school performance and birth size indicators by the categories of family SEP and sex. Girls had better school performance and higher BPI but lower BW and BL than boys. For school performance, the largest SEP gradient was found for maternal education and smallest for household income. BW and BL showed positive gradients for all SEP indicators. For BPI, the SEP gradients were negative but generally weak.

**Table 1:** Percentages of participants and means and standard deviations (SD) of school performance at 16 years of age and weight, length and ponderal index at birth according to childhood family socio-economic position.

	%	School performance (grade 4–10)		Weight (kg)		Length (cm)		Ponderal index (kg/m^3^)	
		Mean	SD	Mean	SD	Mean	SD	Mean	SD
Boys									
Maternal education									
Basic	18	6.88	0.98	3.61	0.56	50.6	2.24	27.8	2.41
Secondary	69	7.37	1.04	3.66	0.53	50.9	2.16	27.8	2.39
Tertiary	12	8.15	0.94	3.67	0.52	50.9	2.12	27.7	2.36
Paternal education									
Basic	24	7.00	1.01	3.62	0.56	50.7	2.24	27.8	2.41
Secondary	62	7.35	1.04	3.66	0.53	50.8	2.15	27.8	2.39
Tertiary	15	8.12	0.95	3.68	0.52	50.9	2.12	27.7	2.36
Household incomes									
Lowest quintile	20	7.17	1.07	3.64	0.54	50.7	2.19	27.8	2.38
2. to 4. Quintile	60	7.31	1.05	3.66	0.54	50.8	2.16	27.8	2.39
Highest quintile	20	7.79	1.04	3.65	0.53	50.9	2.18	27.7	2.39
Girls									
Maternal education									
Basic	18	7.40	1.03	3.48	0.53	49.8	2.14	28.1	2.46
Secondary	70	7.96	1.01	3.53	0.51	50.0	2.04	28.1	2.44
Tertiary	12	8.66	0.83	3.54	0.49	50.1	2.02	28.1	2.42
Paternal education									
Basic	24	7.56	1.05	3.49	0.53	49.8	2.13	28.1	2.47
Secondary	61	7.93	1.02	3.53	0.51	50.0	2.04	28.1	2.44
Tertiary	15	8.60	0.85	3.55	0.49	50.1	2.00	28.1	2.42
Household incomes									
Lowest quintile	20	7.71	1.07	3.52	0.51	49.9	2.07	28.2	2.44
2. to 4. quintile	60	7.89	1.04	3.53	0.51	50.0	2.05	28.1	2.44
Highest quintile	20	8.34	0.96	3.52	0.51	50.0	2.06	28.1	2.44

**Fig. 1:**
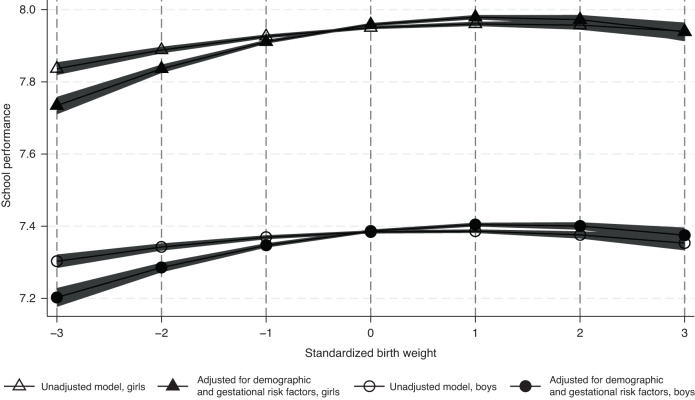
The association between birth weight and school performance at 16 years of age by sex.

We started the statistical modeling by studying the associations between birth size and school performance at the individual level (Table 2). Greater BW and BL were generally associated with better school performance and greater BPI with lower school performance (Model 1). BL explained a larger proportion of variation of school performance (0.08% in boys and 0.15% in girls) than BW (0.02% and 0.05%, respectively) or BPI (0.07% and 0.04%, respectively). Adjusting the results for demographic risk factors strengthened the association for BL but practically eliminated the association for BPI (Model 2). This was mainly because parity was negatively associated with school performance but positively associated with birth size (Supplementary Table 3). For BW, adjusting the results for demographic and gestational risk factors (Model 3) strengthened the association between smaller BWs and school performance. This made the association between BW and school performance more curvilinear as demonstrated in Fig. 1. For BL, adjusting the results for gestational risk factors and family SEP (Model 4) explained a part of the association (32% in boys and 22% in girls) when compared to the results adjusted only for demographic factors (Model 2). For the other birth size indicators, the effect of adjustment was less systematic: for BW, the adjustment for family SEP weakened the association with smaller BWs (i.e., decreased the linear effect) whereas for BPI, the negative association was seen after the adjustment for gestational risk factors, but the further adjustment for family SEP explained a part of this negative association.

**Table 2:** Regression coefficients (β) for school performance with 95% confidence intervals (CI) of standardized birth weight, length and ponderal index in boys and girls.

	Model 1			Model 2			Model 3			Model 4		
	β	95% CI		β	95% CI		β	95% CI		β	95% CI	
		LL	UL		LL	UL		LL	UL		LL	UL
Weight												
Boys												
Linear effect	0.133	0.094	0.171	0.162	0.124	0.199	0.245	0.196	0.295	0.160	0.114	0.207
Quadratic effect	−0.006	−0.008	−0.004	−0.006	−0.008	−0.005	−0.011	−0.013	−0.008	−0.007	−0.009	−0.005
Girls												
Linear effect	0.155	0.114	0.196	0.199	0.159	0.239	0.306	0.254	0.358	0.220	0.171	0.270
Quadratic effect	−0.007	−0.009	−0.005	−0.008	−0.010	−0.006	−0.014	−0.016	−0.011	−0.010	−0.012	−0.007
Sex interaction	0.0032			0.001			0.1111			0.059		
Length												
Boys												
Linear effect	0.031	0.027	0.035	0.041	0.038	0.045	0.038	0.033	0.042	0.028	0.024	0.032
Girls												
Linear effect	0.042	0.038	0.046	0.053	0.049	0.056	0.049	0.044	0.053	0.041	0.037	0.045
Sex interaction	<0.00001			<0.00001			0.0005			0.0001		
Ponderal index												
Boys												
Linear effect	−0.028	−0.032	−0.024	−0.003	−0.007	0.000	−0.009	−0.013	−0.006	−0.005	−0.008	−0.001
Girls												
Linear effect	−0.022	−0.025	−0.018	0.001	−0.003	0.004	−0.007	−0.011	−0.003	−0.006	−0.009	−0.002
Sex interaction	0.016			0.0776			0.1731			0.9009		

Model 1: Unadjusted results; Model 2: adjusted for birth year+maternal age+parity; Model 3: adjusted for Model 2+maternal smoking+ gestational age+square of gestational age+ Apgar score; Model 4: adjusted for Model 3+maternal education+paternal education+household incomes.

Next, we analyzed how the association between BW and school performance was modified by family SEP (Table 3; the interaction parameter estimates are available in Supplementary Table 4). Generally, the effect sizes for both the positive linear and the negative quadratic BW effects were stronger in higher SEP categories in boys and girls when adjusting only for demographic factors (Model 1). This suggests that in higher SEP families, the association between BW and school performance was seen in particular with smaller BWs whereas in lower SEP families the association was seen over a wider range of the BW distribution. This is demonstrated in Supplementary Figs 2–4: where in lower SEP families the association between BW and school performance was nearly linear, in higher SEP families the association was weak and slightly lower school performance was seen only at the smaller end of the BW distribution. Adjusting the results for gestational risk factors strengthened both linear and quadratic effects (Model 2): this suggests that these adjustments made the association between BW and school performance more curvilinear.

**Table 3:** Regression coefficients (β) with 95% confidence intervals (CI) of standardized birth weight according to family socio-economic position in boys and girls.

	Model 1						Model 2					
	Linear effect			Quadratic effect			Linear effect			Quadratic effect		
	β	95% CI		β	95% CI		β	95% CI		β	95% CI	
		LL	UL		LL	UL		LL	UL		LL	UL
Boys												
Maternal education												
Basic	−0.044	−0.119	0.031	0.003	0.000	0.007	0.097	−0.004	0.199	−0.004	−0.009	0.001
Secondary	0.128	0.083	0.172	−0.005	−0.007	−0.003	0.222	0.163	0.281	−0.010	−0.013	−0.007
Tertiary	0.068	−0.037	0.173	−0.003	−0.008	0.002	0.179	0.039	0.320	−0.008	−0.015	−0.001
Interaction	0.0009						0.0006					
Paternal education												
Basic	0.015	−0.052	0.083	0.001	−0.003	0.004	0.078	−0.012	0.169	−0.003	−0.007	0.001
Secondary	0.123	0.075	0.171	−0.005	−0.007	−0.003	0.234	0.171	0.297	−0.010	−0.013	−0.007
Tertiary	0.124	0.031	0.217	−0.006	−0.010	−0.001	0.257	0.131	0.383	−0.011	−0.018	−0.005
Interaction	0.005						0.0184					
Household incomes												
Lowest quintile	0.147	0.064	0.231	−0.005	−0.009	−0.001	0.196	0.086	0.305	−0.008	−0.014	−0.003
2. to 4. quintile	0.126	0.078	0.174	−0.005	−0.007	−0.003	0.226	0.163	0.289	−0.010	−0.013	−0.007
Highest quintile	0.187	0.103	0.270	−0.008	−0.012	−0.004	0.299	0.187	0.411	−0.014	−0.019	−0.008
Interaction	<0.0001						0.0147					
Girls												
Maternal education												
Basic	0.103	0.016	0.190	−0.003	−0.008	0.001	0.260	0.146	0.374	−0.012	−0.017	−0.006
Secondary	0.118	0.071	0.165	−0.004	−0.007	−0.002	0.250	0.187	0.312	−0.011	−0.014	−0.008
Tertiary	0.151	0.055	0.247	−0.007	−0.012	−0.002	0.266	0.137	0.394	−0.012	−0.019	−0.006
Interaction	0.0236						0.0921					
Paternal education												
Basic	0.152	0.075	0.230	−0.005	−0.009	−0.001	0.284	0.182	0.385	−0.012	−0.017	−0.007
Secondary	0.112	0.062	0.162	−0.004	−0.007	−0.001	0.237	0.171	0.303	−0.010	−0.014	−0.007
Tertiary	0.098	0.008	0.189	−0.005	−0.009	0.000	0.202	0.082	0.322	−0.009	−0.015	−0.003
Interaction	<0.0001						0.407					
Household incomes												
Lowest quintile	0.180	0.089	0.272	−0.006	−0.011	−0.002	0.213	0.093	0.332	−0.009	−0.015	−0.003
2. to 4. quintile	0.190	0.139	0.242	−0.008	−0.010	−0.005	0.324	0.257	0.391	−0.014	−0.018	−0.011
Highest quintile	0.166	0.085	0.248	−0.007	−0.011	−0.003	0.290	0.179	0.401	−0.013	−0.019	−0.008
Interaction	0.0002						0.6207					

Model 1: Adjusted for birth year+maternal age+parity; Model 2: adjusted for Model 1+maternal smoking+gestational age+square of gestational age+Apgar score.

We then repeated similar SEP interaction analyses for BL and BPI (Table 4; the interaction parameter estimates are available in Supplementary Table 5). When adjusting the results for the demographic factors, BL systematically showed the weakest positive association with school performance in high SEP families (Model 1). Adjusting the results for the gestational risk factors decreased these SEP gradients, but they were still seen in particular in girls (Model 2). For BPI, no association with school performance was seen in any SEP category when adjusting the results for the demographic factors (Model 1). After adjusting the results for gestational risk factors, weak negative associations with school performance were seen (Model 2). However, these associations did not systematically differ between the SEP categories.

**Table 4:** Regression coefficients (β) with 95% confidence intervals (CI) of standardized birth length and ponderal index according to family socio-economic position in boys and girls.

	Boys						Girls					
	Model 1			Model 2			Model 1			Model 2		
	β	95% CI		β	95% CI		β	95% CI		β	95% CI	
		LL	UL		LL	UL		LL	UL		LL	UL
Length												
Maternal education												
Basic	0.024	0.016	0.031	0.024	0.015	0.033	0.042	0.033	0.050	0.038	0.028	0.047
Secondary	0.032	0.027	0.036	0.036	0.031	0.041	0.042	0.037	0.046	0.049	0.044	0.054
Tertiary	0.011	0.002	0.021	0.022	0.011	0.033	0.011	0.002	0.020	0.027	0.017	0.037
Interaction	0.0272			0.0004			0.0001			0.0015		
Paternal education												
Basic	0.031	0.025	0.038	0.027	0.019	0.035	0.053	0.045	0.060	0.047	0.038	0.056
Secondary	0.032	0.028	0.037	0.035	0.029	0.040	0.042	0.038	0.047	0.047	0.042	0.052
Tertiary	0.013	0.005	0.022	0.027	0.017	0.037	0.012	0.003	0.020	0.027	0.018	0.037
Interaction	0.0074			0.0613			<0.0001			0.0398		
Household incomes												
Lowest quintile	0.056	0.048	0.064	0.039	0.029	0.048	0.065	0.057	0.074	0.047	0.037	0.057
2. to 4. quintile	0.035	0.031	0.040	0.036	0.030	0.041	0.051	0.046	0.056	0.050	0.044	0.055
Highest quintile	0.031	0.023	0.039	0.033	0.023	0.042	0.031	0.023	0.039	0.039	0.030	0.048
Interaction	<0.0001			0.0099			<0.0001			0.1555		
Ponderal index												
Maternal education												
Basic	0.004	−0.004	0.012	0.001	−0.007	0.009	0.004	−0.005	0.012	−0.003	−0.011	0.006
Secondary	−0.006	−0.011	−0.002	−0.010	−0.014	−0.005	−0.004	−0.008	0.000	−0.008	−0.013	−0.004
Tertiary	0.000	−0.010	0.009	−0.001	−0.011	0.009	−0.001	−0.009	0.008	0.000	−0.008	0.009
Interaction	0.0016			0.0202			0.0225			0.0582		
Paternal education												
Basic	−0.004	−0.011	0.003	−0.009	−0.016	−0.001	0.007	0.000	0.015	−0.001	−0.008	0.007
Secondary	−0.002	−0.007	0.002	−0.006	−0.011	−0.001	−0.003	−0.007	0.002	−0.008	−0.012	−0.003
Tertiary	−0.002	−0.011	0.007	−0.003	−0.012	0.006	−0.006	−0.014	0.002	−0.006	−0.013	0.002
Interaction	0.6115			0.3962			0.0035			0.0546		
Household incomes												
Lowest quintile	−0.003	−0.011	0.006	−0.010	−0.018	−0.001	0.004	−0.004	0.013	−0.008	−0.017	0.000
2. to 4. quintile	−0.003	−0.007	0.002	−0.008	−0.012	−0.003	0.001	−0.003	0.006	−0.005	−0.010	−0.001
Highest quintile	−0.001	−0.009	0.007	−0.006	−0.014	0.002	−0.003	−0.011	0.004	−0.007	−0.014	0.001
Interaction	0.7945			0.7666			0.4428			0.9615		

Model 1: Adjusted for birth year+maternal age+parity; Model 2: adjusted for Model 1+maternal smoking+gestational age+square of gestational age+Apgar score.

Finally, we analyzed the association between body size and school performance within families to take into account unmeasured shared family characteristics (Table 5). Within full siblings, the associations between birth size indicators and school performance were weak in the model adjusted only for the demographic factors (Model 1). However, when we adjusted the results for gestational risk factors, we found that BW and BL were associated with school performance in a similar direction as in the individual-level analyses (Model 2). However, the effect sizes were weaker than in the individual-level analyses in boys and girls. When we analyzed these associations within twin pairs, the associations were again in the same direction as in the individual-level and full-sibling analyses. However, because of the more limited sample size, the confidence intervals were wide thus making it difficult to evaluate the effect sizes. As in the individual-level analyses, the association between BPI and school performance was weak, both within full siblings and twins.

**Table 5:** Within family regression coefficients (β) with 95% confidence intervals (CI) standardized birth weight, length and ponderal index in full same sex siblings and twins.

	Boys						Girls					
	Model 1			Model 2			Model 1			Model 2		
	β	95% CI		β	95% CI		β	95% CI		β	95% CI	
		LL	UL		LL	UL		LL	UL		LL	UL
Siblings												
Weight												
Linear effect	0.028	0.046	0.101	0.105	0.009	0.201	−0.001	−0.081	0.079	0.169	0.064	0.273
Quadratic effect	−0.001	−0.005	0.002	−0.004	−0.009	0.000	0.000	−0.004	0.004	−0.007	−0.012	−0.002
Length												
Linear effect	0.004	−0.004	0.012	0.016	0.008	0.025	0.014	0.006	0.022	0.031	0.021	0.040
Ponderal index												
Linear effect	−0.005	−0.012	0.002	−0.003	−0.011	0.004	−0.006	−0.013	0.001	−0.004	−0.011	0.004
Twins												
Weight												
Linear effect	0.075	−0.315	0.465	0.018	−0.380	0.416	0.130	−0.267	0.528	0.129	−0.270	0.528
Quadratic effect	0.000	−0.023	0.023	0.003	−0.020	0.027	−0.005	−0.030	0.019	−0.005	−0.030	0.019
Length												
Linear effect	0.064	0.028	0.100	0.064	0.028	0.100	0.028	−0.008	0.064	0.028	−0.008	0.065
Ponderal index												
Linear effect	0.009	−0.021	0.039	0.008	−0.022	0.039	0.004	−0.026	0.033	0.002	−0.028	0.032

Model 1: Adjusted for maternal age+parity (sibling analyses only); Model 2: adjusted for Model 1+maternal smoking+gestational age+square of gestational age (sibling analyses only)+Apgar score.

## Discussion

In this large longitudinal study of children, we found that BL showed a linear association with school performance in late adolescence, whereas the association between BW and school performance was curvilinear. BL also explained more of the variation of school performance than BW. Even though some previous research has shown a positive association between BL and cognitive performance,^12^ the studies are still rare when compared to the large number of studies analyzing BW.^7,8^ Our results suggest that BL is a better indicator of the prenatal environment than BW within the normal variation of birth size. According to our initial hypothesis, BPI was negatively associated with school performance. However, this negative association was explained by birth order whereas the adjustment for birth order made the association between BW and school performance more curvilinear. This was because higher birth order was associated with lower school performance, as is also well demonstrated in previous studies,^20,21^ but larger birth size. It is possible that there are different mechanisms behind these associations if, for example, family dynamics or social resources explain the negative association between birth order and school performance but physiological changes of the uterus during consequent pregnancies explain the positive association between birth order and birth size. Thus, in contrast to our hypothesis, BPI does not contribute to the curvilinear association between BW and school performance.

Even when BL showed a robust association with school performance, the absolute effect size was modest and BL variation explained only a small proportion of school performance variation (0.08% in boys and 0.15% in girls). This translates to a 0.03 increase of mean grade in boys and 0.04 in girls per 1 SD change in BL. However, even when small, the explained part of school performance variation in our study was still considerably larger than that found between BL and several IQ measures in adulthood (0.02% or less) in a Danish study,^22^ and in both of these studies it was larger than that found for BW. A limitation in the previous Danish study^22^ is that it pooled males and females and thus cannot study sex differences, which was also the case in a previous Norwegian study studying BL that only included males.^12^ Thus, our study suggesting that BL has a stronger effect on further cognitive performance in girls than in boys is unique. The small effect size is expected when considering that BL and BW are only indicators of the prenatal environment rather than causal risk factors of further school performance, as also supported by previous Mendelian randomization studies.^9,10^ This observation is also consistent with our results that twins had lower BL and BW than singletons, but their school performance was nevertheless better. This result is not surprising considering that there are likely to be different mechanisms behind growth restriction in twin and singleton pregnancies.^23^

We obtained more robust evidence on the role of BL as an indicator of environmental risk factors in analyses of how family-level factors modified these associations. Adjusting the results for gestational risk factors and family SEP explained around a third of the association between BL and school performance. Further, the associations of BL and BW with school performance were weaker within families than between individuals supporting the idea that the birth size indicators partly reflect family-level risk factors. However, it is noteworthy that in particular after taking into account gestational risk factors, the association of BL and BW with school performance was also seen within families. This is consistent with previous findings that lower BW is associated with shorter education^24^ and lower IQ^25^ within twin pairs. These results support the idea that the association between birth size and later school performance is not only explained by family background but can also reflect unique risks related to each pregnancy. Our results stratified by family SEP are also consistent with the hypothesis that BL and BW reflect familial risk factors. The association between BL and school performance was stronger in low and intermediate SEP families as compared to high SEP families. Family SEP also modified the shape of the association between BW and school performance: where in low SEP families the association was nearly linear, in high SEP families it was weak and curvilinear, with associations observed only with smaller BWs. Thus, population heterogeneity according to SEP can contribute to the curvilinear association between BW and school performance.

The stronger association of BL than BW with school performance can reflect the changing effects of intra-uterine environmental factors during pregnancy. The fetus gains most weight during the third trimester and most length during the second trimester of pregnancy.^26^ Thus, BL may better reflect environmental conditions during the periods most critical for neurodevelopment. However, this is difficult to determine with any certainty since the human brain also develops during the third trimester.^26^ It is also possible that BW more reflects the natural constraints of the uterus to adjust to the growing fetus than BL. Our results are consistent with this idea since multiple pregnancies, which are more prone to the physical restrictions of the uterus than singleton pregnancies, were more strongly associated with BW than BL, but twins nevertheless had better school performance than singletons (Supplementary Table 1). This hypothesis is also consistent with the results that in girls, who have smaller birth size than boys and are thus less prone to these constraints, BL is more strongly associated with school performance. Even though we cannot empirically evaluate all the possible mechanisms, our results suggest that BL better reflects environmental variations during the most critical phases of pregnancy for cognitive development than BW.

Our data have strengths but also weaknesses. Our main strength is our very large and representative register-based data that also includes girls, which has not been available in some previous large-scale studies utilizing conscription registers. Parental education and income are register based and thus not prone to reporting bias. Our baseline data cover all children born in Finland. However, during the follow-up, we lost around 7% of these children because of early mortality, emigration, drop-out from primary school or not continuing to secondary education. This has probably made our results more conservative. A limitation is that we do not have direct information on IQ or other direct indicators of cognitive performance. Further studies should evaluate whether the association between birth size and school performance are mediated through IQ or whether there are also other mediating mechanisms. Additionally, our anthropometric measures were limited to BW and BL. More detailed anthropometric measures, such as head and waist circumferences and body composition measures, could provide more information on these associations.

In conclusion, BL shows a linear and BW a curvilinear association with school performance in late adolescence. Family SEP modified these associations, which can indicate that the birth size indicators reflect more the intrauterine environment in the presence of low family social resources. The effect sizes are small and thus birth size cannot be used, e.g., for identifying children with particular schooling needs. However, at a population level, BL can offer useful information on the intrauterine environment affecting later-life cognitive development.

### Supplementary information

Supplementary information

## Data Availability

The data that support the findings of this study are available from Statistics Finland but restrictions apply to the availability of these data, which were used under license for the current study, and so are not publicly available. Data are, however, available from the authors upon reasonable request and with permission of Statistics Finland.
