# Supplementary material for: Birth size, school performance and family social position: a study of 650,000 children
Source: Pediatr Res. 2023 Jul 29;94(6):2105–14. doi: 10.1038/s41390-023-02757-1 (PMC10665183; doi:10.1038/s41390-023-02757-1)
Supplement: Supplementary file 1 — Supplementary information [file 41390_2023_2757_MOESM1_ESM.pdf]

Supplementary table 1. The regression coefficients (β) with 95% confidence intervals (CI) of twins and higher order multiples compared to singletons (reference category) for school grade and birth size in boys and girls.

	Boys[1]						Girls[2]					
	Twins			Triplets and higher orders multiples			Twins			Triplets and higher orders multiples		
	β	95% CI		β	95% CI		β	95% CI		β	95% CI	
		LL	UL		LL	UL		LL	UL		LL	UL
School grade (4-10)	0.13	0.10	0.15	0.37	0.25	0.49	0.03	0.00	0.05	0.15	0.03	0.26
Birth weight (kg)	-0.94	-0.95	-0.92	-1.66	-1.72	-1.60	-0.93	-0.94	-0.92	-1.65	-1.71	-1.60
Birth length (cm)	-0.04	-0.04	-0.03	-0.07	-0.07	-0.06	-0.03	-0.03	-0.03	-0.06	-0.07	-0.06
Ponderal index (kg/m^3)	-1.91	-1.96	-1.85	-3.62	-3.90	-3.34	-2.25	-2.30	-2.19	-3.85	-4.12	-3.58

[1]Explained part of variation: 0.04% for school grade, 7.32% for birth weight, 6.24% for birth length and 1.63% for ponderal index.
[2]Explained part of variation: <0.01% for school grade, 8.19% for birth weight, 6.57% for birth length and 2.17% for ponderal index.

Supplementary table 2. Regression coefficients (β) with 95% confidence intervals of school grades by classified birth weight, length and ponderal index.

	Boys			Girls		
	β	95% confidence intervals		β	95% confidence intervals	
		LL	UL		LL	UL
**Weight**						
Lowest decile	ref.			ref.		
2. decile	0.036	0.018	0.054	0.059	0.044	0.074
3. decile	0.041	0.023	0.059	0.081	0.065	0.096
4. decile	0.056	0.038	0.074	0.084	0.068	0.099
5. decile	0.062	0.044	0.079	0.095	0.080	0.111
6. decile	0.065	0.048	0.083	0.097	0.081	0.113
7. decile	0.070	0.053	0.087	0.095	0.078	0.111
8. decile	0.073	0.056	0.090	0.102	0.085	0.118
9. decile	0.059	0.042	0.076	0.090	0.073	0.107
Highest decile	0.040	0.024	0.057	0.073	0.055	0.090
**Length**[1]						
Lowest quintile	ref.			ref.		
2. quintile	0.050	0.038	0.061	0.056	0.046	0.066
3. quintile	0.061	0.050	0.072	0.071	0.060	0.081
4. quintile	0.076	0.064	0.088	0.093	0.081	0.105
Highest quintile	0.091	0.080	0.102	0.096	0.082	0.109
**Ponderal index**						
Lowest decile	ref.			ref.		
2. decile	-0.016	-0.031	0.000	0.005	-0.013	0.022
3. decile	-0.020	-0.036	-0.004	0.017	-0.001	0.034
4. decile	-0.036	-0.052	-0.020	-0.008	-0.026	0.009
5. decile	-0.043	-0.059	-0.027	-0.012	-0.029	0.005
6. decile	-0.037	-0.053	-0.020	-0.020	-0.037	-0.003
7. decile	-0.062	-0.078	-0.045	-0.036	-0.053	-0.019
8. decile	-0.057	-0.074	-0.041	-0.036	-0.053	-0.019
9. decile	-0.083	-0.100	-0.066	-0.048	-0.065	-0.031
Highest decile	-0.097	-0.114	-0.080	-0.059	-0.075	-0.042

[1]Quintiles are used since the measurement of birth length is less precise than for weight.

Supplementary table 3. Regression coefficients (β) with 95% confidence intervals of school grades and birth size indicators by birth order and sex.

	Boys			Girls		
	β	95% confidence intervals		β	95% confidence intervals	
		LL	UL		LL	UL
School grade						
1. pregnancy	-0.175	-0.184	-0.166	-0.165	-0.174	-0.157
2. pregnancy	-0.266	-0.276	-0.255	-0.269	-0.280	-0.258
3. pregnancy	-0.368	-0.385	-0.351	-0.381	-0.398	-0.364
4. or later pregnancy	-0.376	-0.397	-0.356	-0.324	-0.344	-0.304
Birth weight						
1. pregnancy	0.315	0.307	0.323	0.278	0.270	0.286
2. pregnancy	0.388	0.377	0.398	0.341	0.331	0.350
3. pregnancy	0.375	0.359	0.392	0.354	0.338	0.369
4. or later pregnancy	0.443	0.424	0.462	0.428	0.410	0.446
Birth length						
1. pregnancy	0.173	0.165	0.181	0.149	0.141	0.156
2. pregnancy	0.201	0.191	0.211	0.176	0.167	0.186
3. pregnancy	0.152	0.136	0.168	0.160	0.144	0.175
4. or later pregnancy	0.178	0.159	0.198	0.180	0.162	0.199
Birth ponderal index						
1. pregnancy	0.273	0.265	0.281	0.259	0.251	0.267
2. pregnancy	0.343	0.334	0.353	0.325	0.315	0.335
3. pregnancy	0.392	0.376	0.408	0.369	0.353	0.385
4. or later pregnancy	0.464	0.446	0.483	0.463	0.444	0.483

Supplementary table 4. Interaction parameters (β) with 95% confidence intervals (CI) of standardized birth weight according to family socio-economic position in boys and girls.

	Model 1						Model 2					
	Linear effect			Quadratic effect			Linear effect			Quadratic effect		
	β	95% CI		β	95% CI		β	95% CI		β	95% CI	
		LL	UL		LL	UL		LL	UL		LL	UL
**Boys**												
**Maternal education**												
Basic	ref.			ref.			ref.			ref.		
Secondary	0.1821	0.0930	0.2713	-0.0094	-0.0139	-0.0049	0.1673	0.0786	0.2560	-0.0079	-0.0124	-0.0034
Tertiary	0.1508	0.0143	0.2874	-0.0081	-0.0150	-0.0013	0.1460	0.0102	0.2817	-0.0067	-0.0135	0.0001
**Paternal education**												
Basic	ref.			ref.			ref.			ref.		
Secondary	0.1214	0.0386	0.2042	-0.0064	-0.0106	-0.0022	0.1181	0.0359	0.2003	-0.0056	-0.0098	-0.0015
Tertiary	0.1416	0.0213	0.2619	-0.0078	-0.0138	-0.0017	0.1432	0.0237	0.2626	-0.0068	-0.0128	-0.0008
**Household incomes**												
Lowest quintile	ref.			ref.			ref.			ref.		
2. to 4. quintile	-0.0065	-0.1022	0.0891	-0.0007	-0.0055	0.0041	-0.0025	-0.0971	0.0920	-0.0005	-0.0053	0.0043
Highest quintile	0.0791	-0.0389	0.1972	-0.0051	-0.0110	0.0009	0.0964	-0.0203	0.2131	-0.0051	-0.0110	0.0007
**Girls**												
**Maternal education**												
Basic	ref.			ref.			ref.			ref.		
Secondary	0.0369	-0.0596	0.1334	-0.0023	-0.0072	0.0027	0.0101	-0.0856	0.1057	0.0001	-0.0049	0.0050
Tertiary	0.1071	-0.0346	0.2487	-0.0065	-0.0138	0.0008	0.0809	-0.0595	0.2213	-0.0036	-0.0108	0.0036
**Paternal education**												
Basic	ref.			ref.			ref.			ref.		
Secondary	-0.0194	-0.1083	0.0695	0.0001	-0.0045	0.0046	-0.0319	-0.1199	0.0561	0.0014	-0.0032	0.0059

Tertiary	0.0045	-0.1238	0.1327	-0.0021	-0.0087	0.0045	0.0032	-0.1237	0.1300	-0.0007	-0.0072	0.0058
**Household incomes**												
Lowest quintile	ref.			ref.			ref.			ref.		
2. to 4. quintile	0.0330	-0.0689	0.1349	-0.0025	-0.0077	0.0028	0.0469	-0.0534	0.1473	-0.0027	-0.0078	0.0025
Highest quintile	0.0342	-0.0888	0.1572	-0.0032	-0.0095	0.0032	0.0494	-0.0717	0.1705	-0.0029	-0.0091	0.0033

Model 1: Adjusted for birth year+maternal age+parity; Model 2: adjusted for Model 1+maternal smoking+gestational age+square of gestational age+Apgar score

Supplementary table 5. Interaction parameters (β) with 95% confidence intervals (CI) of standardized birth length and ponderal index according to family socio-economic position in boys and girls.

	Boys						Girls					
	Model 1			Model 2			Model 1			Model 2		
	B	95% CI		β	95% CI		β	95% CI		β	95% CI	
		LL	UL		LL	UL		LL	UL		LL	UL
**Length**												
**Maternal education**												
Basic	ref.			ref.			ref.			ref.		
Secondary	0.0051	-0.0037	0.0139	0.0178	0.0090	0.0265	-0.0020	-0.0112	0.0073	0.0147	0.0055	0.0239
Tertiary	-0.0094	-0.0221	0.0034	0.0122	-0.0005	0.0249	-0.0260	-0.0393	-0.0127	0.0015	-0.0117	0.0147
**Paternal education**												
Basic	ref.			ref.			ref.			ref.		
Secondary	-0.0012	-0.0093	0.0070	0.0093	0.0012	0.0174	-0.0126	-0.0212	-0.0041	-0.0007	-0.0091	0.0078
Tertiary	-0.0164	-0.0278	-0.0051	0.0032	-0.0081	0.0145	-0.0383	-0.0503	-0.0262	-0.0138	-0.0257	-0.0019
**Household incomes**												
Lowest quintile	ref.			ref.			ref.			ref.		
2. to 4. quintile	-0.0222	-0.0313	-0.0131	-0.0136	-0.0226	-0.0046	-0.0157	-0.0253	-0.0061	-0.0067	-0.0161	0.0028
Highest quintile	-0.0244	-0.0356	-0.0132	-0.0072	-0.0182	0.0039	-0.0313	-0.0431	-0.0196	-0.0113	-0.0228	0.0003
**Ponderal index**												
**Maternal education**												
Basic	ref.			ref.			ref.			ref.		
Secondary	-0.0158	-0.0250	-0.0066	0.4413	0.3502	0.5324	-0.0115	-0.0204	-0.0025	-0.0051	-0.0139	0.0038
Tertiary	-0.0057	-0.0187	0.0073	0.9735	0.8445	1.1025	-0.0030	-0.0157	0.0097	0.0072	-0.0054	0.0197
**Paternal education**												
Basic	ref.			ref.			ref.			ref.		
Secondary	-0.0033	-0.0117	0.0051	0.0009	-0.0075	0.0093	-0.0138	-0.0220	-0.0055	-0.0098	-0.0179	-0.0017

Tertiary	0.0008	-0.0108	0.0124	0.0074	-0.0041	0.0189	-0.0135	-0.0248	-0.0021	-0.0046	-0.0158	0.0066
**Household incomes**												
Lowest quintile	ref.			ref.			ref.			ref.		
2. to 4. quintile	-0.0031	-0.0125	0.0063	-0.0017	-0.0110	0.0076	-0.0051	-0.0142	0.0040	-0.0007	-0.0096	0.0083
Highest quintile	-0.0014	-0.0129	0.0101	0.0016	-0.0097	0.0130	-0.0067	-0.0179	0.0044	0.0005	-0.0104	0.0115

Model 1: Adjusted for birth year+maternal age+parity; Model 2: adjusted for Model 1+maternal smoking+gestational age+square of gestational age+Apgar score

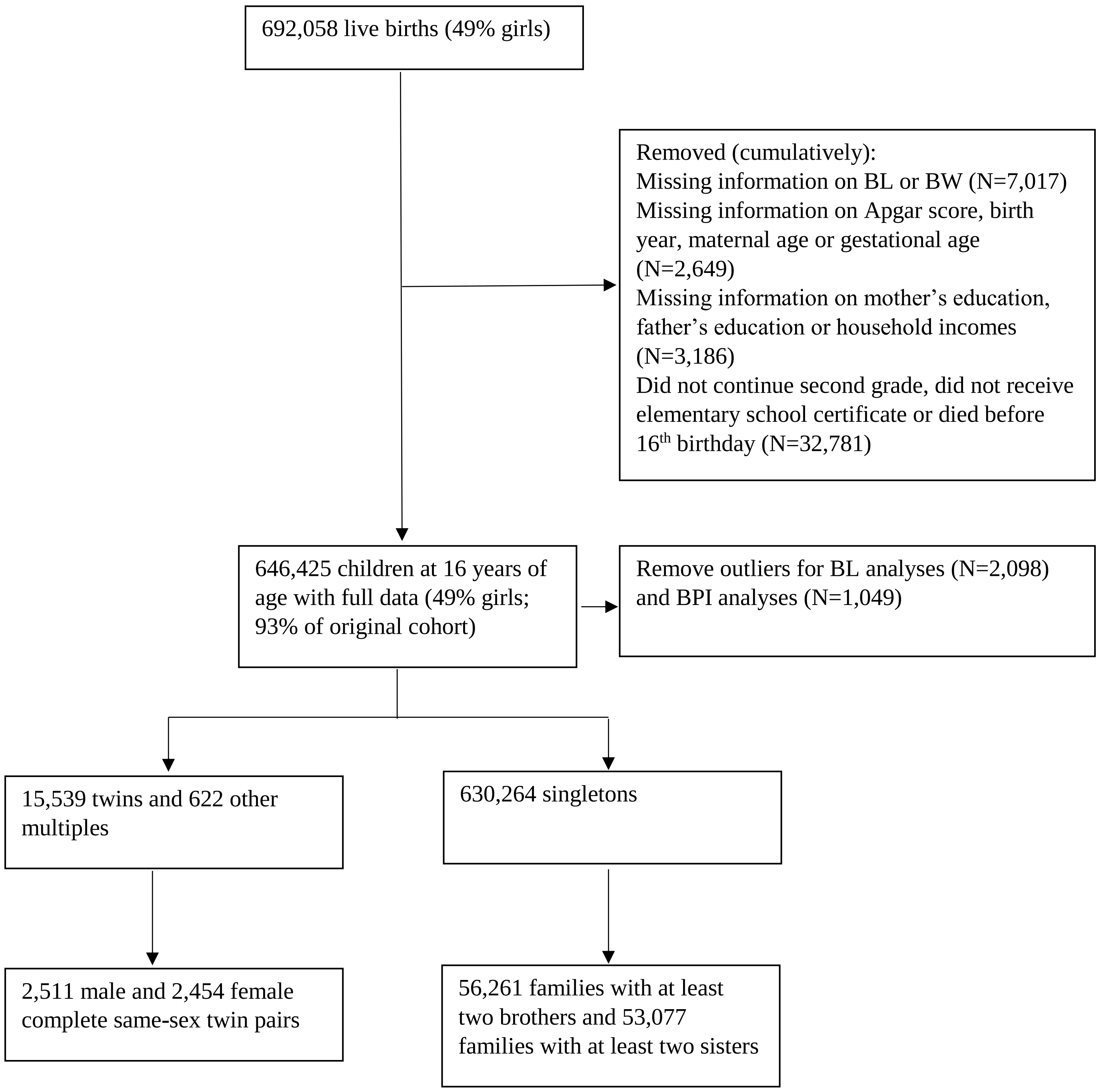

Supplementary figure 1. Flow diagram of the study cohort.

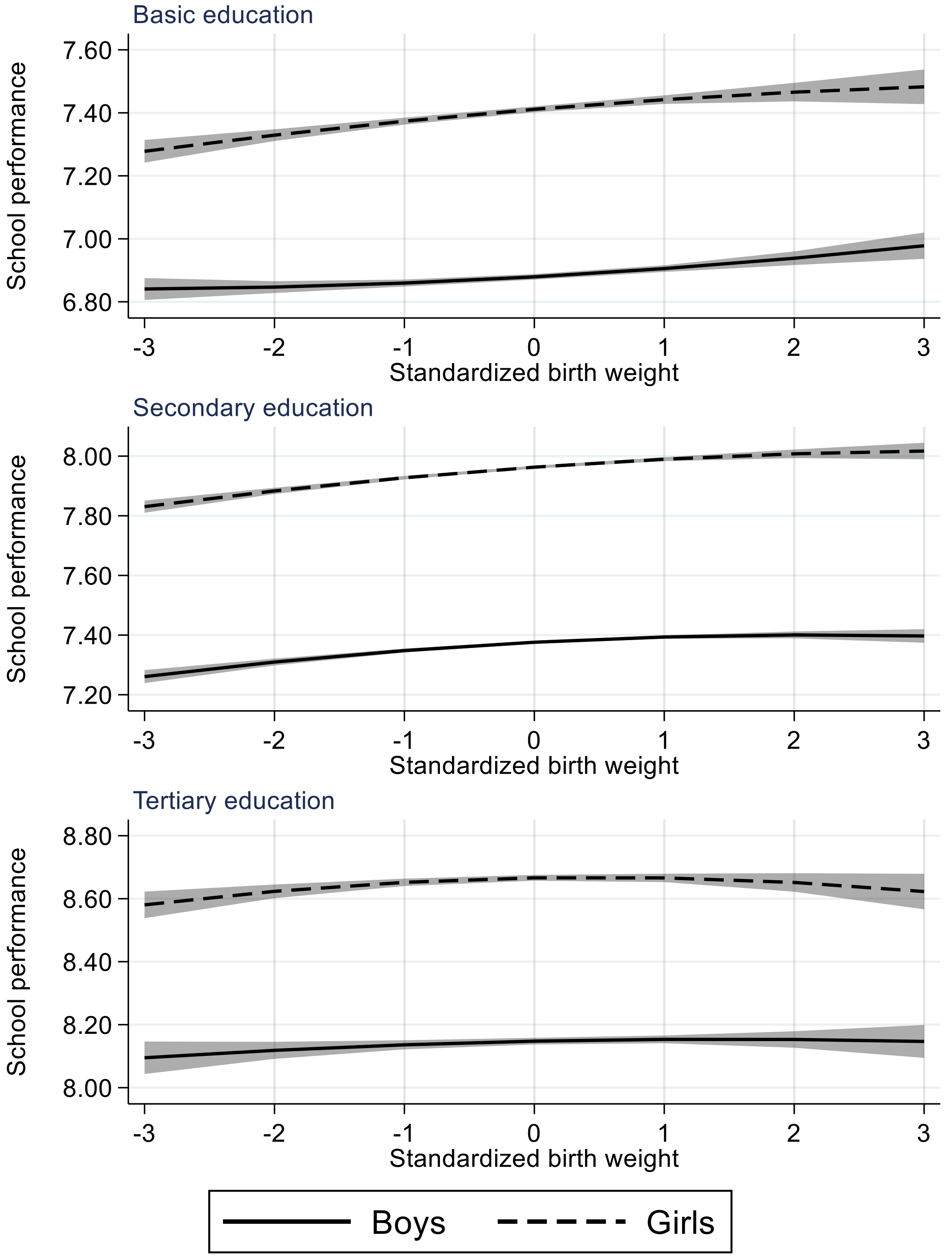

Supplementary figure 2. The association between birth weight and school performance at 16 years of age by sex and maternal education.

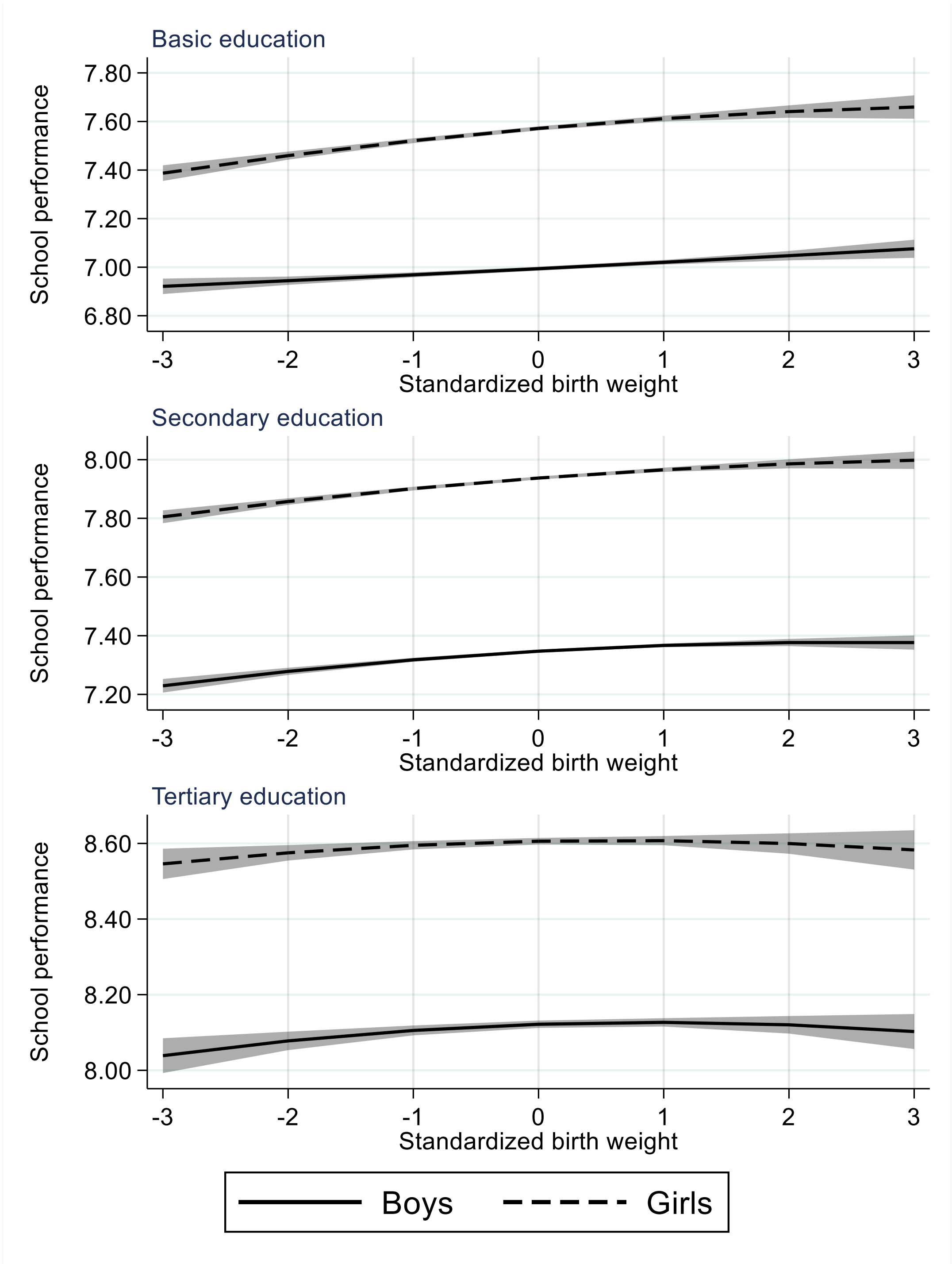

Supplementary figure 3. The association between birth weight and school performance at 16 years of age by sex and paternal education.

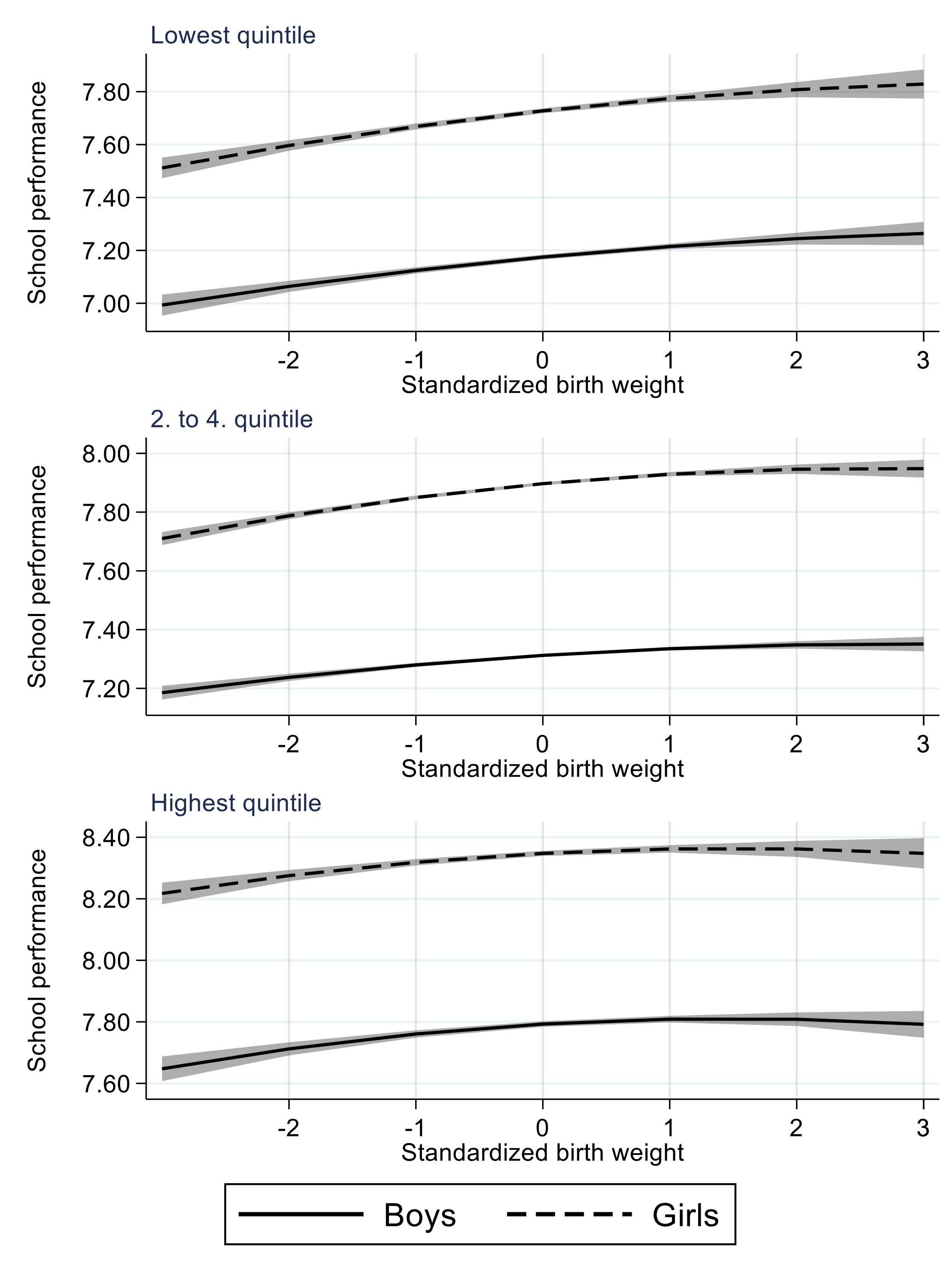

Supplementary figure 4. The association between birth weight and school performance at 16 years of age by sex and family incomes.
